# Arabidopsis 2010 and beyond—big science with a small weed

**DOI:** 10.3389/fpls.2013.00018

**Published:** 2013-02-13

**Authors:** Klaus Harter, Andreas P. M. Weber

**Affiliations:** ^1^Department of Plant Physiology, Center for Plant Molecular Biology, University of TübingenTübingen, Germany; ^2^Center of Excellence on Plant Sciences, Department of Biology, Institute for Plant Biochemistry, Heinrich Heine UniversityDüsseldorf, Germany

Over the past two decades revolutionary progress in plant biology became possible by focusing resources on a single plant reference system, *Arabidopsis thaliana*. After the completion of the Arabidopsis genome sequence in the year 2000, a coordinated multinational effort was launched to “determine the function of every gene in Arabidopsis” by the year 2010.

Part of this effort was the German Arabidopsis Functional Genomics Network (AFGN). Established in 2001, AFGN was continuously supported for 9 years by the German Research Foundation (DFG). Over 85 German researchers contributed to AFGN, partially in close bilateral collaboration with scientists of the NSF-funded Arabidopsis 2010 initiative.

While the ambitious goal of determining the function of every Arabidopsis gene has not yet been fully achieved, the Arabidopsis genome is now one of the best annotated and serves as the gold standard for plant and other genomes. A large and international community has established novel methods, toolkits and genomic resources, such as sequence-indexed mutant collections and comprehensive and easily accessible “omics-scale datasets,” ranging from transcriptome over proteome to the metabolome. One prominent example is the AtGenExpress data set, which was partially realized by AFGN scientists and serves as the gold standard in microarray-based transcriptomics.

The Arabidopsis 2010 program evolved from studying the functions of single genes and gene families to comprehensive systems-wide analyses of functional networks, thereby paving the way from descriptive to predictive plant science. Progress does not stop here—in the near future, the genomes of 1000 Arabidopsis strains and accessions will become available, which will make it possible to exploit existing natural variation for addressing fundamental questions in ecology and evolutionary biology in an unprecedented manner. Further, due to ease of transformation and existing genetic and genomic resources, Arabidopsis will likely serve as a chassis for synthetic plant biology, an emerging field and challenge for the next decade of plant research (EU 2020 Vision of Plant Science, 2008; An International Model for the Future of Plant Science, 2010).

This special issue of Frontiers in Plant Physiology will provides 20 examples from the ongoing research of AFGN and Arabidopsis 2010 members on how focusing on a single plant model system has impacted and revolutionized many fields of plant research and it will provide an outlook on the upcoming challenges and fields of research for the next decade of Arabidopsis research.
